# Hand Sanitizer Intoxication in the Emergency Department

**DOI:** 10.7759/cureus.17906

**Published:** 2021-09-12

**Authors:** Ali Pourmand, Mateen Ghassemi, Sarah E Frasure, Alexandrer Kreisman, Robert Shesser

**Affiliations:** 1 Department of Emergency Medicine, George Washington University School of Medicine and Health Sciences, Washington DC, USA

**Keywords:** alcohol misuse, substance abuse, hand sanitizer, hand hygiene, ethanol intoxication

## Abstract

Hand hygiene has always been an area of emphasis within the hospital setting, never more so than during the coronavirus disease 2019 (COVID-19) pandemic. The consumption of alcohol-containing hand sanitizer products, whether intentional or accidental, often garners attention, particularly since these products may contain methanol. This report describes a case of surreptitious theft and intentional ingestion of the emergency department’s (ED) ethanol-based hand sanitizer by a patient who presented to the ED clinically intoxicated with a high ethanol level. When the patient remained clinically intoxicated for more than 18 hours and had a rising serum ethanol level in the ED, clinicians searched his belongings and found several purloined bottles of the ED’s hand sanitizer. When confronted, the patient admitted to ingesting hand sanitizer during his ED stay. This case highlights the need for clinicians to be suspicious of intentional ingestion of ethanol-containing products for at-risk patients. Additionally, it demonstrates that measures and protocols should be put in place that minimize the ability for the inappropriate use of these widely accessible products within the hospital.

## Introduction

Non-beverage or surrogate alcohols are substances that may be consumed as an alternative to traditional alcoholic beverages containing alcohol. These substances may include mouthwash, perfumes, aftershave, cough syrup, liquid cold remedies, rubbing alcohol, gasoline, paint thinner, jellied alcohols, hairspray, and hand sanitizers [1-4]. Some individuals suffering from alcoholism may choose these methods of alcohol consumption in order to become intoxicated. The widespread availability of hand sanitizers during the coronavirus disease 2019 (COVID-19) pandemic has led to an increase in the intentional ingestion of ethanol-based hand sanitizers.

Per World Health Organization and Centers for Disease Control guidelines for hand hygiene, alcohol-based hand sanitizers are readily available in healthcare settings [5,6]. Used as low-viscosity rinses, gels, or foams, alcohol-based hand sanitizers contain 60-95% ethanol or isopropanol and are efficacious for reducing the transmission of pathogenic microorganisms [7]. However, their accessibility and presence in the emergency department (ED) poses a hazard for patients with alcohol dependency as they may be tempted to imbibe ethanol-based hand sanitizers. Although accidental or intentional ingestion of hand sanitizers by children and teenagers have received media attention and have been previously discussed in the literature [8], we present a case in which an adult patient, who requested to be transferred to a detoxification program while in the ED, surreptitiously ingested hand sanitizer while under clinical supervision.

## Case presentation

A 30-year-old male with a history of ethanol use disorder (consuming 1.5 L/day of liquor) complicated by withdrawal seizures, depression, and asthma presented to the ED via emergency medical services after being found intoxicated in a park. Initial vital signs were notable for blood pressure 146/79 mmHg, heart rate 118 beats/minute, respiratory rate 20 breaths/minute, temperature 98.6°F, and oxygen saturation of 99% on room air. His physical examination, aside from being intoxicated with slurred speech and an unsteady gait, was unremarkable. There was no external evidence of trauma. Given his risk of self-reported withdrawal seizures and delirium tremens, the patient was placed on a telemetry monitor and on the Clinical Institute Withdrawal Assessment for Alcohol (CIWA) protocol. The patient received 2 liters of normal saline fluid and intravenous lorazepam. His initial blood alcohol level (BAL) was 359 mg/dl. The patient initially requested to be transferred to a local program for alcohol detoxification but the requirement for eligibility was a BAL less than 100 mg/dl. The patient was re-evaluated for sobriety during changes of shift by the oncoming emergency medicine resident. Strangely, however, he remained clinically intoxicated after 18 hours in the ED. He was subsequently found to have several bottles of the hospital’s hand sanitizer in his possession. At this time, a repeat BAL was as 372 mg/dl and the patient was admitted to the hospital 20 hours after his initial arrival to the ED for observation and monitoring for possible development of withdrawal symptoms.

## Discussion

Although cases of intentional hand sanitizer ingestion have been previously described, we describe a unique case in which a patient, who voluntarily requested to enter a detoxification program, did not disappear or hide to secretly ingest hand sanitizers [9-11]. Instead, while waiting to be transferred to the Psychiatric Institute of Washington for detoxification, the patient ingested ethanol-based hand sanitizer from dispensers around the ED-an environment in which patients and clinicians are constantly in close contact. Previous cases report hospitalized patients drinking hand sanitizer in their rooms or in the bathroom [12-16]. Other settings also included the psychiatric unit, a homeless shelter bathroom, or a prison [17]. This case demonstrates the ease by which a patient suffering from ethanol use disorder can obtain alcohol in the emergency department.

Although ingestion of ethanol-based hand sanitizers should follow the general treatment protocol as ethanol consumption, intoxication from ethanol-based hand sanitizers can lead to serious and life-threatening adverse health effects. These include, but are not limited to, central nervous system and respiratory depression, cardiac dysrhythmias or arrest, nausea and vomiting, liver injury, and lactic and ketoacidosis [18]. Protection of the patient’s respiratory function with possible intubation is a priority given ethanol’s effect on depressing the central nervous system [19,20].

Currently, there are no recommendations regarding the potential misuse of hand sanitizers. Given the extensive utilization of hand sanitizers, coupled with a surge in ethanol sales since the beginning of the COVID-19 pandemic, healthcare providers should be aware of the potential misuse of hand sanitizers as intoxicants. The widespread endorsement of hand sanitizers by public health and medical experts opens a new dimension in medicine, one in which clinicians must also be cautioned of their potential misuse by patients suffering from alcoholism or other mental illnesses. We recommend that emergency staff be educated regarding the possibility of high-risk patients imbibing hand sanitizer, as well as signs of intoxication or changes in mental status. Clinicians should be well-versed in asking specific questions to patients or family members regarding potential alcohol-containing solutions in the household, such as engine additives stored in the garage or mouthwash in the bathroom.

Hospitals should implement measures or protocols that minimize the possibility of the misuse of ethanol-based hand sanitizers. Such protocols include a reevaluation of where hand sanitizer dispensers are physically located throughout the emergency department and their accessibility to patients at higher risk. Since hand sanitizer bottles or pouches in wall dispensers can be easily removed and consumed by patients, we recommend the installation of lockable wall dispensers or the use of alcohol-based wipes. This recommendation would not deter from the practice of using the hand sanitizer when entering or leaving the patient’s room to prevent the transmission of pathogens. Lastly, hand sanitizer dispensers often have the percent alcohol content written on the bottle which may appeal to patients with alcohol dependence and result in consumption. One case report described a patient who secretly drank isopropanol-based hand sanitizer in the bathroom of a hospital because the label indicated “63% v/v isopropyl alcohol,” believing it had higher alcohol content than vodka [20]. Thus, it may be advisable to adjust hand sanitizer labels to make the alcohol content less recognizable to patients, and decrease attraction for abuse. The installation of additional sinks with soap dispensers would be another option in high-risk areas in hospitals or other inpatient facilities.

Promoting and maintaining hand hygiene while also protecting patients with alcohol abuse disorders presents a major challenge to both patient safety and risk mitigation. Clinicians should be aware of the possible misuse of hand sanitizer. Increased awareness and implementation of safety protocols in emergency departments might minimize intentional hand sanitizer ingestion. We recommend the use of lockable wall dispensers as a way to reduce hand sanitizer ingestion in the hospital.

## Conclusions

Promoting and maintaining hand hygiene while also protecting patients with alcohol abuse disorders presents a major challenge to both patient safety and risk mitigation. Clinicians should be aware of the possible misuse of hand sanitizer. Increased awareness and implementation of safety protocols in emergency departments might minimize intentional hand sanitizer ingestion. The use of lockable wall dispensers or alcohol-based wipes could help to reduce hand sanitizer ingestion in the hospital.
